# Acute intrathoracic gastric volvulus: A rare delayed presentation of congenital diaphragmatic hernia: A case report

**DOI:** 10.1016/j.ijscr.2020.04.066

**Published:** 2020-05-11

**Authors:** Mostafa Zain, Mohamed Abada, Mohamed Abouheba, Ahmed Elrouby, Amir Ibrahim

**Affiliations:** Department of Pediatric Surgery, Faculty of Medicine, University of Alexandria, Egypt

**Keywords:** Gastric volvulus, Diaphragmatic hernia, Organoaxial rotation, Case report

## Abstract

**Introduction:**

Acute intrathoracic gastric volvulus occurs when the stomach undergoes organoaxial rotation in the chest due presence of a diaphragmatic defect. Gastric volvulus associated with congenital diaphragmatic hernia is extremely rare and can be explained as 2 of the 4 ligaments supporting the stomach (gastrophrenic and gastrosplenic) which are connected to the left diaphragm may become elongated or absent. According to the current literature, only 27 pediatric cases have been reported so far.

**Presentation of case:**

We describe an 8 years old boy who presented to our emergency department with acute epigastric pain and vomiting. The chest radiograph obtained in the emergency department demonstrated an elevated gastric air-fluid level in the left hemithorax. A computed tomography scan demonstrated a sizable left diaphragmatic defect admitting stomach, small bowel loops and transverse colon with organoaxial gastric volvulus. Emergent laparotomy was done for reduction of the viscera to the abdominal cavity and repair of the diaphragmatic defect. The patient showed an uneventful recovery without experience any pain or difficulty with eating.

**Discussion:**

Acute gastric volvulus is a rare pathology defined as an abnormal rotation of the stomach for more than 180° leading to a closed-loop obstruction which may progress to ischemia and strangulation. A plain standing abdominal X-ray and an upper gastrointestinal contrast study are useful for diagnosis, but some authors recommend performing CT or MRI to confirm the diagnosis. The standard treatment for volvulus is open laparotomy with detorsion and anterior gastropexy.

**Conclusion:**

The presented case highlights congenital diaphragmatic hernia associated with gastric volvulus is a serious condition with very high morbidity and mortality. It should be considered in the differential diagnosis of children with epigastric pain and uncontrolled non bilious vomiting. An upper gastrointestinal contrast study is useful for early diagnosis and surgical treatment should not be delayed awaiting further complementary imaging tests.

## Highlights

• Acute gastric volvulus occurs when the stomach undergoes torsion in the thoracic cavity.
• We describe an 8-year-old boy with diaphragmatic hernia complicated by gastric volvulus presented with acute epigastric pain and non-bilious vomiting.
• He underwent laparotomy. Reduction of the viscera to the abdominal cavity, gastropexy and repair of diaphragmatic defect were done.

## 1 Introduction

The term “volvulus” is a Latin verb known as volvere, which means to turn or roll [1]. Intrathoracic gastric volvulus in children is a rare surgical emergency. It occurs when the stomach rotates more than 180 degrees which creates a closed-loop obstruction of the foregut that can result in incarceration and strangulation. The stomach is fixed at the esophageal hiatus and the pylorus by many ligaments. The stretching of these ligaments or their absence can result in gastric volvulus [2].

Etiology of gastric volvulus is either primary or secondary. In primary cause which accounts for 30% of gastric volvuli, there is absence of diaphragmatic defects or intra-abdominal abnormality causing the volvulus. Causes of secondary gastric volvulus are many as; congenital or traumatic diaphragmatic defects, abdominal bands or hiatal hernias [3].

Gastric volvulus in children is a difficult diagnosis to reach as children always present with nonspecific clinical symptoms. Mortality due to acute gastric volvulus is as high as 42–56%, secondary to gastric ischemia and perforation [4]. In this article, we report a case of an 8 years old boy with acute intrathoracic gastric volvulus which is a rare delayed presentation of diaphragmatic hernia. The work has been reported in line with the SCARE criteria [5].

## 2 Case report

**Fig. 1:**
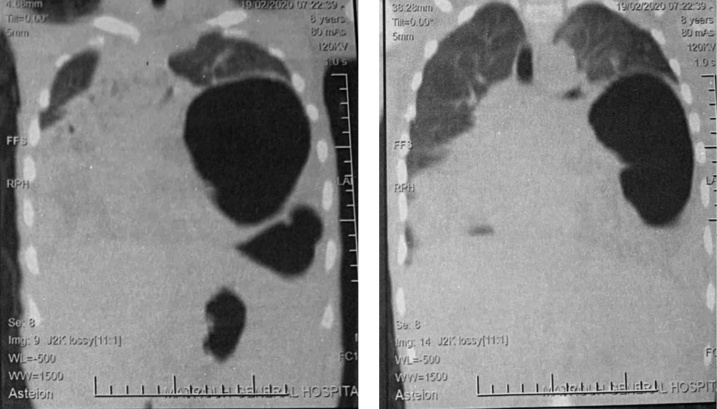
CT scan with coronal reconstruction showing distended stomach, due to organoaxial gastric volvulus.

An 8 years old boy presented to our emergency department with an acute episode of severe epigastric pain, and uncontrolled non bilious vomiting. Upon arrival, physical examination revealed diffuse tenderness over the epigastric area with hyperactive bowel sounds. The chest radiograph obtained in the emergency department demonstrated an elevated gastric air-fluid level in the left hemithorax. A computed tomography scan demonstrated a sizable left diaphragmatic defect measuring 4 cm and admitting stomach, small bowel loops and transverse colon with organoaxial gastric volvulus. It showed also that the stomach was severely distended due to organoaxial volvulus with antrum and pylorus superior to fundus leading to gastric outlet obstruction (Fig. 1).

**Fig. 2:**
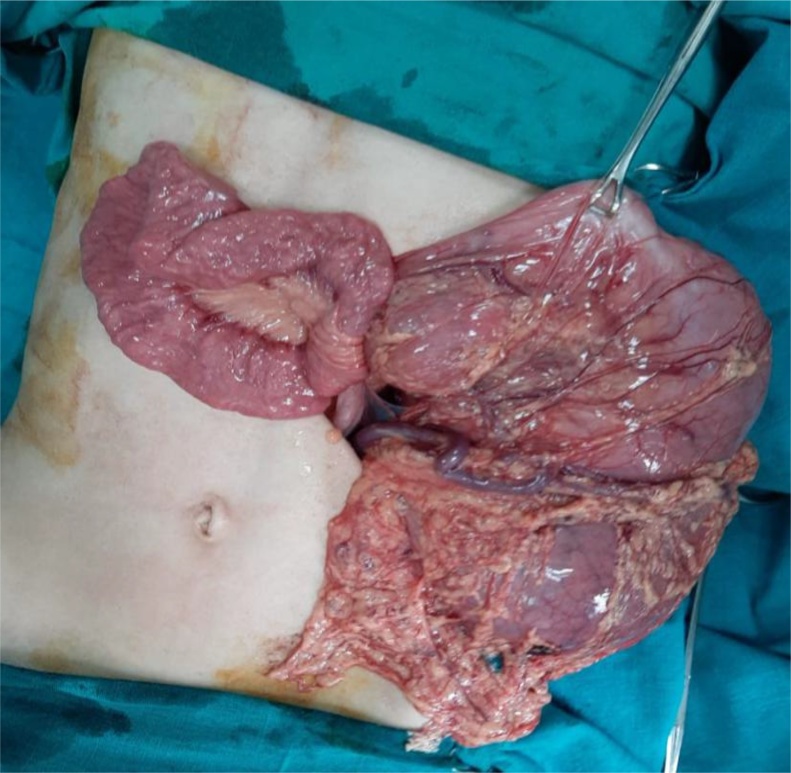
Intraoperative findings: the stomach is rotated with displacement of the cardia under the pyloric level.

The patient was admitted to the Pediatric Intensive Care Unit (PICU) for resuscitation with IV fluids to correct dehydration before surgery. Through a left subcostal incision, exploration revealed a left diaphragmatic defect measuring approximately 4 cm admitting stomach, small bowel loops and transverse colon. The stomach was congested and hugely dilated requiring deflation by a nasogastric tube before it can be reduced to the abdomen. It appeared to be twisted as an organoaxial volvulus with antrum and pylorus superior to fundus (Fig. 2). Reduction of the herniated viscera to the abdomen was done with direct closure of the defect using silk 0 sutures. Then, gastropexy was done in 3 points to the anterior abdominal wall using silk 2/0 sutures. A left chest tube was inserted to drain the fluid collection in the chest.

The patient showed an uneventful recovery, started oral feeding on the 2^nd^ post-operative day, the chest tube was removed on the 3^rd^ post-operative day and was discharged from the hospital on the 5^th^ post-operative day.

## 3 Discussion

Acute gastric volvulus is a rare pathology which was firstly described in pediatric population by Oltmann in 1899 [6]. It is defined as an abnormal rotation of the stomach for more than 180° leading to a closed-loop obstruction which may progress to ischemia and strangulation [7]. According to the axis by which the stomach rotates upon itself, Singelton classified gastric volvulus into 3 types; mesenteroaxial, organoaxial, or combined with the first being the most common type of secondary volvulus in children in contrary to the primary cases where organoaxial predominates [8]. In contrary to that, our case showed organoaxial rotation although being secondary volvulus.

Approximately, 75% of the affected patients present with a triad of symptoms known as “Borchardt triad” including acute localized epigastric distension, unproductive retching, and inability to pass a nasogastric tube [9]. Hematemesis can also be seen due to mucosal sloughing secondary to ischemia or mucosal tear following retching [10].

A plain standing abdominal X-ray and an upper gastrointestinal contrast study are useful for diagnosis, but some authors recommend performing CT or MRI to confirm the diagnosis [11]. However, if the diagnosis is clear with plain X-ray contrast studies, surgical treatment should not be delayed with further complementary imaging tests.

The principal aims of surgery include reduction of the volvulus, prevention of recurrence and repairing any predisposing factors as diaphragmatic defects [12]. Although open surgical reduction with gastropexy was considered to be the standard treatment [4], as with most procedures, advancements in laparoscopic instruments and refinement of laparoscopic techniques have enabled more complex procedures to be performed using minimally invasive techniques. Less invasive techniques currently employed include laparoscopic surgery or endoscopic reduction with insertion of a percutaneous gastrostomy tube in patients with isolated gastric volvulus [13,14]. In our case, the patient was hemodynamically unfit for laparoscopy. So, we resorted to open laparotomy. Derotation of the volvulus can be made easier after decompressive puncture of the dilated stomach or decompression using NG tube [15], as done in our case.

Gastric volvulus associated with CDH is extremely rare and can be explained as 2 of the 4 ligaments supporting the stomach (gastrophrenic and gastrosplenic) which are connected to the left diaphragm may become elongated or absent. According to the current literature, only 27 pediatric cases have been reported so far [16].

To conclude, congenital diaphragmatic hernia associated with gastric volvulus is a serious condition with very high morbidity and mortality. It should be considered in the differential diagnosis of children with epigastric pain and uncontrolled non bilious vomiting. An upper gastrointestinal contrast study is useful for early diagnosis and surgical treatment should not be delayed awaiting further complementary imaging tests.

## Funding

The authors received no financial support for the research, authorship, and/or publication of this article.

## Ethical approval

This case report has been approved by local ethics committee at Alexandria University Hospitals according to the declaration of Helsinki.

## Consent

Parental written informed consent on behalf of the patient was obtained for publication of this case report and accompanying images. A copy of the written consent is available for review by the Editor-in-Chief of this journal on request.

## Author contribution

Concept – Zain; Design – Zain; Supervision – Abouheba; Materials – Abada; Data Collection and Processing – Ibrahim; Analysis and Interpretation – Ibrahim; Writer – Zain; Critical Review – Elrouby.

## Registration of research studies

NA.

## Guarantor

Mostafa Zain.

## Provenance and peer review

Not commissioned, externally peer-reviewed.

## Declaration of Competing Interest

The authors declared no potential conflicts of interest with respect to the research, authorship, and/or publication of this article.
